# Insights into the Molecular Mechanisms Behind Intralesional Immunotherapies for Advanced Melanoma

**DOI:** 10.3390/cancers12051321

**Published:** 2020-05-22

**Authors:** Dejan Vidovic, Carman Giacomantonio

**Affiliations:** Department of Surgery, Faculty of Medicine, Dalhousie University, Halifax, NS B3H 4R2, Canada; dejan.vidovic@dal.ca

**Keywords:** in-transit melanoma, intralesional, intratumoral, interleukin-2, BCG, T-VEC, rose bengal

## Abstract

The incidence of cutaneous melanoma, a highly malignant skin cancer, is increasing yearly. While surgical removal of the tumor is the mainstay of treatment for patients with locally confined disease, those with metastases face uncertainty when it comes to their treatment. As melanoma is a relatively immunogenic cancer, current guidelines suggest using immunotherapies that can rewire the host immune response to target melanoma tumor cells. Intralesional therapy, where immunomodulatory agents are injected directly into the tumor, are an emerging aspect of treatment for in-transit melanoma because of their ability to mitigate severe off-target immune-related adverse events. However, their immunomodulatory mechanisms are poorly understood. In this review, we will summarize and discuss the different intralesional therapies for metastatic melanoma with respect to their clinical outcomes and immune molecular mechanisms.

## 1. Introduction

Cutaneous melanoma is one of the most common forms of cancer today, ranking fifth most common in men and seventh most common in women [1]. Melanoma also carries a high mortality rate; in 2019, an estimated 7800 individuals will be newly diagnosed with melanoma, and 1300 will die from the disease [2]. Overall prognosis is closely linked to the staging of the tumor, where more advanced cases with nodal involvement or metastasis have 5-year observed survival rates ranging beginning at 78% and dropping to 15% as tumor burden increases [3].

The incidence of melanoma has been one of the most rapidly increasing over the last several decades and is projected to continue that trend moving forward [1,4,5]. Consequently, the overall cost and economic burden of melanoma to healthcare systems is hypothesized to drastically increase [1,6]. From a primary prevention point of view, these costs can be mitigated through adequate public health policy implementation (i.e., promoting reduced UV exposure) [1]. However, from a tertiary care point of view (i.e., treating patients who already have melanoma), discovery and utilization of highly cost-effective therapies is paramount [7].

Initial therapies for early stages of melanoma often begin with excisional surgery followed by lymph node dissection, if appropriate [8]. While most patients (up to 84%) present with such localized disease, a minority of patients, approximately between 9% and 4%, present with regional or distant metastatic disease (stage III+ disease), respectively [9]. Prognosis for these patients is generally poor, as their tumors are often disseminated and unresectable; and unlike other solid tumors, advanced melanoma typically responds poorly to traditional chemotherapy [8]. For those patients with stage III in-transit disease or greater, current guidelines recommend enrolment in a clinical trial or treatment with intralesional or systemic immunotherapies [8], which have been developed to exploit the immunogenicity inherent to melanoma. This review will examine all of the established intralesional therapies used for treatment of advanced melanoma, with a specific focus on the immunological and biochemical mechanisms behind them, and how those affect treatment outcomes.

## 2. Immunological Basis of Melanoma

Melanoma is widely considered an immunogenic cancer, able to arise largely due to an evasion of immunosurveillance. This is eloquently illustrated by its 2–8 fold increased incidence in organ transplant recipients who are chronically immunosuppressed [10]. During melanomagenesis, immunoediting becomes predominant, with initial and prolonged elimination of tumor cells via immunosurveillance (a stage that is continuous over years or decades), equilibrium between tumor cells and immune cells, and then, finally, the escape of tumor cells from the immune system [11,12]. Anti-tumor immune responses in the context of melanoma largely follow a pattern of initial innate immune activation mediated by macrophages, granulocytes, and dendritic cells (DCs), all driving a subsequent Th1 response (as opposed to a Th2 response) characterized by activity of melanoma-specific effector CD4^+^, CD8^+^, and γδ T cells, functioning primarily through interferon gamma (IFNγ) [12,13]. During the escape phase, melanoma cells acquire deficient antigen presentation machinery, masking them from the immune system. In combination with release of immunosuppressive cytokines (such as IL8 and IL10) that recruit T regulatory suppressor cells (Tregs) and myeloid-derived suppressor cells (MDSCs), the tumor microenvironment becomes overall a pro-tumorigenic and anti-immune environment [12]. Thus, to overcome this pro-tumorigenic environment, intralesional therapies must adequately promote immune-mediated cytotoxicity and downregulate immunosuppressive mechanisms.

## 3. Intralesional Therapies

### 3.1. Interleukin-2

Interleukin-2 (IL2) is a pleotropic cytokine produced primarily by CD4^+^ T cells following antigen stimulation, but also by CD8^+^ T cells, and to a lesser extent, natural killer (NK) cells and activated DCs [14,15]. The primary function of IL2 is to bind IL2 receptors (IL2R) present on T cell subsets to alter their transcriptional landscape of cytokine receptors and transcription factors [14]. Key factors include granzyme B (GrB), perforin, IFNγ, and transcription factor T-bet, which ultimately drive Th1 cell effector function [14].

As of 2016, intralesional IL2 is supported by NCCN clinical practice guidelines as an effective treatment for in-transit non-resectable melanoma [8]. A major benefit of intralesional IL2 is the mitigation of the undesirable and often severe side effect profile of high-dose systemic IL2 while achieving high doses of IL2 at the tumor site. Indeed, the side effects of intralesional IL2 are modest, with the most common being inflammation at the injection site, mild flu-like symptoms (i.e., fatigue), and nausea. In very rare cases (<10%), headache, rigors, and stomach pains may also occur, but these tend to be mild and respond well to over-the-counter remedies [16,17,18]. Grade 3 or 4 toxicities are exceedingly rare and are isolated to individual cases [8].

There is a paucity of clinical evidence evaluating the use of intralesional IL2 in melanoma. To date, a small handful of studies have been reported using intralesional IL2 alone for metastatic melanoma [16,17,19,20,21]. These early-phase studies vary widely in their IL2 dosage and injection frequency. Nevertheless, they have shown complete response (CR) rates ranging from 32% to 96% (Table 1) [11,12,13,14,15,16]. In general, the data is inconclusive as to whether more aggressive dosing regimens are associated with a higher percentage of CR; for example, the study reporting a 32% CR rate injected an equivalent of <10 million international units (MIU) of IL2 weekly [19], while elsewhere, a 96% CR rate was achieved with anywhere from 3–15 MIU per week [21]. Expectedly, a major negative prognostic indicator (i.e., strongly predicting stable disease or disease progression following intralesional IL2 treatment) is the presence of visceral metastases or stage IV disease [16,17,19]. Additionally, the limited data suggests that intralesional IL2 efficacy tapers as lesions exceed 2 cm in diameter [21]; however, the number of in-transit metastases present does not seem to affect overall response (OR) for an individual patient (i.e., the rate of complete response was similar between those with >20 lesions and those with <20 lesions) [20]. Overall, 76% of in-transit metastases were resolved using intralesional IL2 therapy [20]. Still, a subset of patients with treatment-refractory disease, late-stage disease, or with recurrent metastases may benefit from intralesional IL2 therapy as suggested by a number of case reports showing complete resolution and remission of advanced melanoma [22,23,24].

The exact mechanism of action of intralesional IL2 is unclear, as the scarcity of data limits our understanding; however, there is likely an anti-tumor T cell response that triggers clearance of malignant melanocytes. The limited data available support this hypothesis (summarized in Table 2). In patients with CR, immunohistochemical staining of their lesions reveals no tumor cells with the presence of a CD3^+^ T cell-rich lymphocytic infiltrate supplemented with monocytes, macrophages, and melanophages [17], indicating a potent immune-mediated reaction had occurred. Indeed, another study identified that patients with a complete response had a significantly enhanced CD8^+^ T cell infiltrate [19]. In samples collected one-week post-treatment initiation, there was a significant upregulation of caspase-3 in tumor cells with a loss of Melan-A staining [17]; whether this is a form of immunogenic cell death is inconclusive. Some evidence suggests the lack of a systemic tumor-specific immune response, as distant untreated metastases did not exhibit any response to treatment [16]. However, profiling of one patient’s peripheral blood revealed a significant increase in CD8^+^ T cells specific to the tumor-specific antigen MAGE-A3 during treatment, which continued to increase 2 months after treatment [16], suggesting the possibility of local or mild immune memory. Overall, however, further mechanistic insight into the role of intralesional IL2 in the tumor microenvironment of melanoma is required.

### 3.2. Bacillus Calmette-Guerin

Bacillus Calmette-Guerin (BCG) is a live attenuated bacterial vaccine containing *Mycobacterium bovis*, a common pathogen causing bovine tuberculosis in cattle. The vaccine was originally designed to protect against *Mycobacterium tuberculosis* in humans. While not commonly used for vaccination in the Western world today, BCG is one of the first and most extensively studied immunotherapies [27]. For over 40 years, it has been used as a principle immunotherapy for bladder cancer, where it is highly effective at treating carcinoma in situ and preventing recurrence [62,63].

The use of BCG as an immunotherapy in melanoma also dates back over four decades. Early studies revealed that melanoma lesions injected with intralesional BCG, and in some cases, distant untreated lesions, effectively regressed [64,65]. Indeed, epidemiological studies conducted within the last few decades have revealed a reduced risk of melanoma in patients vaccinated with BCG, supporting its use as a potentiator of immune surveillance and anti-tumor activity [66]. Nevertheless, pooled analyses of trials evaluating the use of BCG as an immunotherapy for melanoma revealed less than favourable results; in addition to having no effect in stage I and II melanoma, BCG only achieved an average CR rate of 10.3% (ranging from 0% to 33%) across all studies which reported that outcome measure [67]. Additionally, numerous major immune-related toxicities were identified in some patients, such as: disseminated granulomatous disease [68,69,70,71]; replacement of melanoma lesions with granulomas [65]; ulceration, necrosis, and abscess formation [72]; severe thrombocytopenia [73]; disseminated intravascular coagulation [26]; and severe anaphylaxis [74], which could prove to be fatal [75]. For these reasons, despite still being recommended as a possible intralesional therapy option for in-transit melanoma in clinical practice guidelines [8], its use has largely fallen out of clinical practice.

Prior to falling out of use, the mechanism underlying the immunotherapeutic effect of BCG in melanoma was unclear. With the advent of new technologies, research into intralesional BCG has made a resurgence in recent years [76] (summarized in Table 2). In addition to acting in its capacity as a vaccine to induce a potent local immune response, evidence suggests BCG can induce a strong shift in the melanoma microenvironment. In one study, after treatment with intralesional BCG, a CD3^+^-rich infiltrate was present in both treated lesions and untreated metastatic lesions [25]. Importantly, the treated lesions showed significantly higher numbers of γδT cells, likely due to higher levels of γδT cell-recruiting chemokines CXCL9, CXCL10, and CXCL11 (signaling via the high levels of CXCR3 on γδT cells) present within the microenvironment of lesions post-treatment [25]. These γδT cells are of the Vδ2 subtype [25,77]; incidentally, one of the most potent activators of Vδ2 γδT cells is (E)-4-hydroxy-3-methyl-but-2-enyl-pyrophosphate (HMB-PP), a known by-product of *Mycobacterium* species [78]. The moderate efficacy (of 10 patients treated, half had a clinical response [25]) of intralesional BCG observed may in part be explained by the finding that most Vδ2 γδT cells infiltrating melanoma tumours have an effector function [79], capable of immediately releasing cytokines and exhibiting cytotoxic properties [80]. Indeed, the γδT cell-produced cytokines significantly elevated in these patients’ tumors post-treatment with intralesional BCG included IFNγ, TNFα, TNFβ, and IL15 [25]. The more typical αβ T cell response expected with most immunotherapies is also absent, as only lowly active αβ T cell (i.e., secreting exceedingly low doses of IFNγ) were present in treated and untreated tumors [25]. Together, this data suggests that intralesional BCG mainly functions via an innate-like immune response.

Additional recent findings also support the notion that intralesional BCG in melanoma functions as an atypical potentiator of innate-like immune responses. Most reports have identified M2-like macrophages as pro-tumorigenic in the context of melanoma, where they inhibit anti-tumor T cell responses and promote tumorigenesis [81]. However, intralesional BCG treatment dramatically alters the transcriptional landscape of M2-like macrophages in the context of melanoma, making them more closely resemble anti-tumorigenic cells, as evidenced in their significantly decreased IL10 production (a pro-tumorigenic cytokine) and increased IL12 production (a Th1-stimulating cytokine) [27]. Furthermore, these altered M2-like macrophages were able to cause a significant increase in the levels of IFNγ produced from co-cultured CD4^+^ T cells, and also significantly increased granzyme B release from CD8^+^ T cells in response to tumor cells [27]. Thus, intralesional BCG is able to also stimulate the adaptive arm of the immune system through transcriptional modulation of innate immune system macrophages.

The data above, combined with the numerous reports of severe immune-related toxicities, suggests intralesional BCG functions as a potent immune activator with functions in both the innate and adaptive immune systems. A recently discontinued phase I study (trial number: NCT01838200) emphasized the ability of intralesional BCG to potentially over-activate the immune system in the context of in-transit melanoma. The study authors hypothesized that BCG could provide some level of immune sensitization in patients who were non-responders to immune checkpoint inhibition. Thus, study participants were first treated with high-dose intralesional BCG until a local immune response developed and were then moved to treatment with the immune checkpoint inhibitor ipilimumab (anti-CTLA4). This combination lead to extreme immune-related adverse events in two of five study participants (the rest of the study participants also experienced grade 1 adverse events); this was precipitated by high concentrations of autoantibodies to both self and tumor antigens [28]. In this scenario, where anti-CTLA4 therapy is often regarded as “removing the brakes”, high-dose intralesional BCG could be seen as “a green light”, ultimately leading to unopposed immune toxicity.

While the above combination therapy proved to be highly ineffective, intralesional BCG combined with topical imiquimod, a toll-like receptor 7 (TLR7) agonist, showed much more promise. In a small-scale retrospective study, nine patients with in-transmit melanoma were first treated with intralesional BCG to activate an immune response, followed by maintenance therapy with topical 5% imiquimod cream. The combination therapy had a 56% CR rate, with the remainder of the patients achieving surgical complete response by surgical excision of remaining persistent lesions [29]. Of the nine patients, seven of them showed no evidence of disease present at follow-up (range 12–49 months); the remaining two passed away from other causes [29]. Indeed, a follow up case report of three additional patients treated with intralesional BCG and 5% imiquimod revealed similar success to the first trial, with all three patients responding to some degree and experiencing tumor regression [30]. The highly favourable effect of imiquimod is possibly due to its ability to modulate intratumoral macrophages in melanoma [82], thus potentiating BCG-induced macrophage plasticity. Additionally, TLR7 activation in melanoma is associated with significant increases in: intratumoral CD8^+^ T and CD4^+^ T cells [53]; anti-tumor macrophages, B cells, IFNγ, IFNα/β, and plasmacytoid dendritic cells [54], and pro-inflammatory cytokines IL6 and IL12 [55]. Overall, TLR7 induction suppresses the metastatic potential of melanoma through inducing a Th1-like response [83]. Taken together, these data strongly suggest that combining imiquimod and intralesional BCG initiates a potent anti-tumor immune response in melanoma.

### 3.3. Intralesional IL2 and Topical Creams

While there is a paucity of studies evaluating the use of intralesional IL2 alone, as noted above, others have supplemented intralesional IL2 with immune modulators to varying degrees of success. Indeed, the relatively high response observed with imiquimod supplementation discussed above is not unique to BCG. An early-phase I/II study using initial sensitization with topical imiquimod followed by intralesional IL2 in 13 patients yielded an overall clinical response rate of 50.5%; of these, 80% were CRs [31]. Intralesional IL2 likely potentiates the Th1-promoting ability of TLR7 activation, which is supported by data revealing that the peripheral CD4^+^/CD8^+^ T cell ratio following treatment with imiquimod and intralesional IL2 is significantly increased in peripheral blood mononuclear cell samples (PBMC) of melanoma patients [56]. This is consistent with a shift in the Th1/Th2 balance favouring a Th1 response, which is evident in the increase in production of IFNγ and loss of Th2-like cytokine IL5 [56]. Importantly, these data suggest a systemic effect in the anti-tumor immune response with the addition of imiquimod; the uncertain lack thereof, as previously discussed, is a possible pitfall of intralesional IL2 monotherapy [16].

Strikingly, further supplementation of intralesional IL2 + imiquimod with retinoid creams achieved a 100% CR in treatment of melanoma metastases [32]. In this case, the dose of intralesional IL2 was significantly higher than previously reported (22 MIU twice weekly until regression occurred). In most of the patients, the side effects reported were relatively mild; local erythema, inflammation, and pain were most common. One patient reported vomiting, nausea, flu-like symptoms, and sclerotic lesions at the injection site that improved approximately a month after discontinuation of therapy [32]. A follow-up case report of a patient with chronic recurrent in-transit metastatic melanoma treated with the same regimen showed a CR with complete removal of the cutaneous melanoma [34]. Indeed, further retrospective case studies of 11 patients treated with high-dose intralesional IL2, imiquimod, and retinoid cream also showed a 100% CR with relatively minor toxicities [33]. In further mechanistic studies, lesions treated with the combination therapy had significantly increased expression of a myriad of pro-inflammatory Th1-like cytokines, notably: IFNγ, IL6, TNF, and IL2Rα [57]. Additionally, expression of Th1-promoting transcription factors T-bet, STAT4, and STAT1 were significantly increased, while both Th2-promoting transcription factor GATA3 and Th17-promoting transcription factor RORC were significantly decreased [57]. Akin to the findings reported with intralesional IL2 and imiquimod alone [56], the peripheral CD4^+^/CD8^+^ T cell ratio was significantly increased, with a specific expansion of both activated T lymphocytes and memory T cell populations [57]. These findings, together with the observation that all of the patients suffered some vitiligo (an immune-mediated attack on melanocytes [84]), support the notion of a systemic immune response against aberrant melanocyte antigens.

The exact role of retinoid supplementation in the above combination therapy is unclear. Retinoids have long been known to induce a plethora of genes involved in immunity, differentiation, and tumor signaling [85,86,87,88,89]. In melanoma, retinoid signalling is known to inhibit mitogen-activated protein kinase (MAPK) signalling, leading to a suppression of proliferation [90]. Numerous studies have also revealed that retinoid signalling is able to induce terminal differentiation of melanoma cancer stem cells, the cells theorized to be responsible for melanoma tumorigenesis and metastasis [91,92,93,94]. The immunomodulating role of retinoids in melanoma is mainly through recruitment of CD8^+^ T cells and suppression of both immunosuppressive MDSCs and macrophages in the tumor microenvironment [95,96]. Given that the presence of MDSCs is one of the poorest prognostic indicators in melanoma patients [97], this result is of utmost importance. Retinoids also significantly increase local expression of IFNγ, GrB, and other Th1-promoting cytokines, as well as major histocompatibility class 1 (MHCI) expression of antigens on melanoma cells [95], further supporting that retinoids play an important role in maintaining a CD8^+^ T cell-driven immune response. Interestingly, the immunostimulatory effect of retinoids was diminished in the absence of CD8^+^ T cells [95], suggesting that the addition of retinoids to a treatment regimen would only be beneficial if CD8^+^ T cells were already primed in the tumor microenvironment, which is likely the major role of intralesional IL2 in this combination therapy.

### 3.4. Intralesional IL2 and Checkpoint Inhibitors

The use of systemic checkpoint inhibitor monotherapy (such as ipilimumab) yields only marginally increased overall survival (from approximately 10 months to 11.4 months) for patients with advanced melanoma, compared to standard-of-care [98]. Akin to the study discussed above with BCG, some have theorized that priming the local immune system with intralesional IL2 would increase efficacy of systemic checkpoint inhibitors for the treatment of metastatic melanoma. Results have been mixed, however; in one study, treatment of 15 patients with the combined therapy (9 MIU of intralesional IL2 twice weekly for 4 weeks and systemic ipilimumab 4 times over 12 weeks) yielded no objective responses in any of the 15 patients, with serious grade 3/4 adverse events in 40% of them [35]. In patients with resistance (i.e., progressive disease) to systemic PD-L1 checkpoint inhibitor monotherapy, subsequent intralesional IL2 therapy was able to induce a CR in 33% of patients with a potent increase in CD4^+^ and CD8^+^ cells from the tumor microenvironment infiltrate [36]. Whether this observed clinical response is at all dependent on previous PD-L1 inhibitor therapy is unclear, as the CR following intralesional IL2 treatment is largely similar to intralesional IL2 monotherapy CR rates.

Contrarily, a small-scale study evaluating the concurrent combined use of intralesional IL2 and systemic pembrolizumab (anti-PD-1 antibody) reported overall positive benefits in two patients, both with CR to the treatment [99]. The purported mechanism is through IL2-mediated sensitization of the tumor microenvironment to increase PD-L1 expression and increase CD8^+^ T cell infiltration, thus increasing the anti-PD-1 treatment efficacy. Unfortunately, drawing these conclusions from such a small sample number is troubling; the observed responses from the combination therapy are not dissimilar from intralesional IL2 monotherapy reported above. Furthermore, the similar treatment regimen discussed above [35] was unable to elicit any clinical response in 15 patients, obfuscating the possible benefit of adding systemic checkpoint inhibition.

In stark contrast to the above studies, administering both ipilimumab and IL2 as intralesional injections over a longer time period was able to increase the CR rate to 67%, with up to 89% of patients experiencing an objective response in untreated lesions [37]. Importantly, the combined intralesional injections yielded mostly minor toxicities, similar to those seen with intralesional IL2 alone (i.e., injection site reaction, flu-like symptoms, fatigue, etc.) [37]. In many of the patients who experienced objective responses, there were systemic increases in Th1-induced cytokines IFNγ, GrB, T-bet, and perforin [37]. These data suggest that systemic immune checkpoint inhibition may not be necessary for successful treatment of melanoma, thus mitigating the associated grade 3/4 toxicities. Indeed, localized checkpoint inhibition has been shown to deplete Treg populations [58], which may aid in generation of systemic anti-tumor immune responses.

### 3.5. Other Intralesional IL2-Based Combination Therapies

Others have attempted to develop novel molecular approaches to intralesional IL2 therapy with mixed success. In one approach, a recombinant form of IL2 was generated wherein the cytokine was fused with the single chain variable fragment of the antibody L19, which normally recognizes the extra-domain B (EDB) of fibronectin, present at sites of angiogenesis [38,100]. This approach would ideally concentrate the drug at the neovascular melanoma microenvironment, increasing its half-life. However, intralesional treatment with L19-IL2 (injected once per week for 4 weeks) revealed a 25% CR rate with approximately 20% of patients sustaining a long-lasting response [38]. At the mechanistic level, treatment caused significant increases in Tregs and in total CD4^+^ T cells, with temporary increases of natural killer (NK) cells; circulating MDSCs were significantly decreased [38]. While the decrease in systemic MDSC numbers is promising, the increase in Tregs is less than ideal. It is plausible that this is due to L19-IL2 accumulation at sites of neovasculature, where IL2-dependent Tregs reside and promote angiogenesis [101,102]. Whether this L19-IL2-induced Treg expansion is sufficient to dampen the potential anti-tumor immune response is unclear. Furthermore, previous evidence using unmodified intralesional IL2 showed that NK cells remained pericapillary following treatment [17], so it is not surprising that a form of IL2 accumulating at vascular sites would increase NK cell numbers. Additional research should identify whether Treg-depleting pre-treatment could improve anti-tumor immune responses induced by intralesional L19-IL2.

### 3.6. Interferon Gamma

Interferon gamma (IFNγ), as discussed above, is a cytokine secreted by activated T cells and other lymphocytes (NK T cells, B cells), that plays a pleiotropic role in the immune system. Through inducing gene expression changes, it mediates a plethora of host immune responses; for example, immunosurveillance, differentiation of CD8^+^ T cells, and orchestration of anti-tumor immunity [39]. It was originally hypothesized that due its ability to mediate such a strong adaptive immune response, IFNγ would be an excellent candidate for early gene therapy. To that end, it was incorporated into various viral vectors and tested in early trials [46,103]. In one early trial, an adenoviral vector expressing IFNγ was injected intralesionally into 11 patients—while no maximal tolerated dose was reached, several grade 3 toxicities were noted. Although clinical efficacy was not an endpoint, five patients experienced what would likely be a PR (minor decrease in tumor size), while just one patient who received injection and follow-up surgical intervention remained disease-free at the time of publication; all of the other patients’ disease progressed [103].

Further research into intralesional IFNγ for advanced melanoma was scarce for years, until a small-scale study using a melanoma vaccine (containing class I MHC-restricted melanoma neoantigens) bolstered with IFNγ was completed [40]. The authors hypothesized that IFNγ would enhance the immunogenicity of the neoantigens injected intralesionally, and permit chemotaxis of T cells and generation of systemic immunity. While levels of CXCL10, CCL5, CXCL11, and circulating T cells were indeed increased just after one intralesional injection, persistent anti-tumor immune responses were not observed. Tumors paradoxically had less immune cell infiltration, and anti-tumor immune response pathways were not upregulated [40]. Thus, the role of IFNγ as an intralesional therapy is unclear.

### 3.7. Talimogene Laherparepvec

Talimogene laherparepvec (T-VEC) is an intralesional oncolytic virus therapy derived from modified herpes simplex virus type 1, with deletion of the virulence genes ICP34.5 and ICP47, causing tumor-selective replication and permissive antigen presentation, respectively [41]. In place of these genes, it expresses granulocyte-macrophage colony-stimulating factor (GM-CSF), a cytokine capable of inducing stem cells to differentiate into granulocytes (eosinophils and neutrophils) and monocytes. Additionally, GM-CSF is able to cause antigen-presenting cells (such as monocytes) to differentiate into dendritic cells, which promotes presentation of local antigens to lymphocytes. In the case of melanoma, this may aid in generation of systemic anti-tumor responses [44]. T-VEC is the most clinically developed and explored intralesional therapy for advanced melanoma. Indeed, early phase II trials using T-VEC for the treatment of metastatic melanoma revealed favourable results, with mild adverse events and a durable OR rate of 26%, with responses in both injected and uninjected lesions, suggesting a systemic anti-immune response [41] (summarized in Table 1). A plethora of subsequent phase II/III studies evaluating the use of T-VEC in advanced melanoma yielded similar or superior results, with OR rates in the range of 26–56.5%, and CR rates ranging from 12–43% [42,43,45,59,60,104,105,106] (summarized in Table 1). In many patients with CRs, the response is quite durable and survival is significantly increased [43,45,104,106]. Even in patients with adverse prognostic indicators (i.e., stage IVM1a/b disease, visceral metastases, multiple co-morbidities), T-VEC was able to produce a durable OR rate of 40% [59]. Furthermore, T-VEC was highly successful in the treatment of melanoma in prior transplant patients with an altered immune landscape [47], further supporting its ability to induce a potent anti-tumor immune response. Indeed, in patients who were treatment refractory to multiple immunotherapies, T-VEC was able to induce CRs and promote systemic immunity [48,107].

As observed in early studies on T-VEC [41], others have also identified systemic immune effects induced via intralesional T-VEC in melanoma—which is vital for reducing disease progression. Aside from the excellent CR rates discussed above, at the lesion level, evidence strongly suggests T-VEC promotes resolution of uninjected lesions. In cutaneous head and neck melanoma, which historically has poor prognosis, a 29.5% CR rate was bolstered by 7.9% and 10.8% response in uninjected non-visceral and visceral lesions, respectively [43]. In non-specific unresected stage IIIB-IV melanoma, T-VEC caused a >50% reduction in size of 34% of uninjected non-visceral lesions and 15% of visceral lesions, with a complete resolution seen in 22% and 9% of the respective lesions [42]. In addition, potent, specific anti-melanocyte immune responses occur following T-VEC administration, as evidenced by the presence of vitiligo in some patients [45].

Mechanistically, studies have revealed that T-VEC treatment causes a significant shift in the tumor microenvironment, with infiltration of activated CD8^+^ T cells expressing both perforin and GrB; some of these T cells also displayed a memory effector phenotype, important for promoting systemic immunity [49] (summarized in Table 2). Additionally, T-VEC suppresses immunosuppressive cell populations, with a significant reduction in both intratumoral Tregs and MDSCs following T-VEC treatment compared to untreated controls [49]. Importantly, T cells derived from peripheral blood and from uninjected lesions in patients treated with T-VEC were both antigen-specific (to Melan-A) and able to produce IFNγ [49], providing further evidence for the development of systemic immunity. Interestingly, intratumoral T cells also had increased expression of PD-1 [49,50]; which suggests that anti-PD-1 therapy could augment the response rates of T-VEC therapy.

### 3.8. T-VEC and Checkpoint Inhibition

Indeed, the most recent approaches have been evaluating the use of systemic checkpoint inhibitors with intralesional T-VEC as a means to increase treatment efficacy. The data support this hypothesis, as combination therapy with pembrolizumab (anti-PD-1 therapy) and T-VEC induced durable OR rates of 62%, with a CR rate of 33% [50]. Uninjected non-visceral lesions and visceral lesions decreased by >50% in 43% and 33% of patients, respectively [50]. Often, response to immunotherapy (especially checkpoint inhibitor therapy) is dependent on a high baseline CD8^+^ T cell infiltration; in the case of T-VEC and pembrolizumab combination therapy, no such dependence was observed [50]. Expectedly, combination treatment induced a significant increase in a plethora of intratumoral immune cell populations: IFNγ-producing CD8^+^ T cells, CD4^+^ T cells, B cells, and memory effector T cells, in both injected and uninjected lesions [50]. Peripherally, combination treatment induced expansion of CD8^+^ T cells. Thus, with the increased intratumoral and circulating levels of PD-1 on T cells induced by T-VEC, the combination therapy benefits greatly from the addition of pembrolizumab [50]. Along a similar principle, combination of ipilimumab with T-VEC was also able to bolster efficacy to an OR of 50%, with a durable CR rate of 22% [51]. Unfortunately, the addition of checkpoint inhibitors to T-VEC may cause some patients to experience grade 3/4 adverse events classically associated with checkpoint inhibition, such as vomiting, fatigue, rash, arthralgia, and in very rare cases, autoimmune hepatitis, aseptic meningitis, pneumonitis, and cytokine-release syndrome [50,51,52]. Together these data strongly suggest that augmentation of T-VEC with checkpoint inhibition, albeit likely expensive and risking serious adverse events, is clinically a viable therapeutic option with excellent potential for locoregional and systemic disease control.

### 3.9. Rose Bengal

Rose Bengal (RB) is a photosensitizing dye that has been used for decades in tests of hepatic function and as a stain for ocular ulcers. Early preclinical studies revealed its ability to induce cell death in melanoma cells through a number of mechanisms [61], which lead to its investigation as an intralesional therapy for melanoma (summarized in Table 2). The clinical agent, PV-10, (10% w/v solution of RB in saline, injected intralesionally) was able to induce a CR in 36% of the injected lesions, with an OR rate of 55% in patients with stage III+ melanoma [108] (Table 1). The excellent tolerability of PV-10 lends itself to a dose-dependent response rate, where increased doses of intralesional therapy increase objective response rates [108]. Sequential treatments with PV-10 in a single center study produced a CR rate of 42% and OR rate of 87% in patients with in-transmit melanoma [109]; promising results for a relatively simple treatment with negligible side-effects. Indeed, retrospective analyses in other centers show a similar trend, with another center reporting an OR rate of 68% and CR rate of 26%, with minimal toxicities [110]. In a major phase II study, the data again supported the robustness of intralesional PV-10 in the treatment of metastatic melanoma, with a CR rate of 50%, and some evidence of systemic immunity as evidenced by a 26% CR rate of uninjected lesions [111].

The mechanism of anti-melanoma PV-10 activity has been extensively interrogated in preclinical studies. PV-10 is able to induce antigen-specific CD8^+^ T cells to proliferate and move to draining lymph nodes (DLN), with some evidence of an increase in T effector memory subsets post-treatment, evident in both injected lesions and uninjected lesions [112]. PV-10 also increased antigen uptake into dendritic cells, with subsequent trafficking of dendritic cells to the DLNs to mature. Consistent with previous reports [61], most melanoma-specific cytotoxicity was via necrosis, with a small amount of apoptosis. The main product of this necrotic cell death is extracellular release of high mobility group box 1 (HMGB1), a hallmark of immunogenic cell death and the anti-tumor immune response [113], and a vital contributor to dendritic cell activation in this context [112]. Importantly, examination of the serum of metastatic melanoma patients treated with intralesional PV-10 revealed significantly higher levels of HMGB1 post-treatment compared to pre-treatment [112], indicating a systemic immunogenic response. Interestingly, when the photosensitized dye is applied to melanoma cells and exposed to natural sunlight, cell death occurs via increased oxidative stress inducing p53-mediated apoptosis [114]. Whether this suggests exposure of lesions to natural sunlight post-treatment would be beneficial to patients is unclear. Nevertheless, the data above make for a strong case for increased study into the potential benefit of Rose Bengal in the treatment of melanoma and other solid tumors.

## 4. Conclusions

The management of advanced melanoma is an ever-evolving field with numerous immunological treatment modalities being explored. Naturally immunogenic tumors (i.e., those with high tumor burden) may readily be treated with single-agent immunotherapies [115]. However, many immunotherapeutic strategies focus on systemic treatments, such as using systemic immune checkpoint inhibitors. These capitalize on established tumor-host immune responses, thus unbridling a cytotoxic cascade that leads to tumor clearance.

However, systemic unbridling of the immune system leads to unacceptable immune-related adverse events (irAEs), even in the context of melanoma therapy [28,35,50,51,52] (Table 1). This is likely a consequence of inappropriate facilitation of host immune activation by non-cancerous, weak host antigens that generate autoimmune responses to host cells, causing significant irAEs that can affect almost any organ system [116,117]. As shown here, the challenge associated with irAEs can be largely mitigated using intralesional approaches [8,16,17,18,32,33,37,41] (Table 1). Targeted intralesional and local delivery of immunomodulatory agents allows for high concentrations of drug at the tumor site, permitting intense host immunomodulation to occur preferentially to tumor antigen present within the tumor microenvironment. Thus, systemic toxicity is blunted; it seems to be limited to the effects of the cytokine response associated with the anti-tumor immune response, and not to a systemic activation of autoimmune T cells. Importantly, some evidence suggests that intralesional approaches may be able to induce potent systemic anti-tumor immune responses [16,22,23,24,29,41,48,57,107], likely due to tumor antigen-primed cytotoxic T cells that home towards the chemokine storm induced by the host-tumor immune reaction.

Antigenicity is a vital factor in establishing a strong anti-tumor immune response. While strongly immunogenic tumors may respond well to checkpoint inhibition, weakly immunogenic tumors are more likely to benefit from immune modulation that results in expression or exposure of neoantigens at the tumor site. Intralesional therapies, such as T-VEC, BCG, PV-10, and IL2 can promote neoantigen exposure (through viral antigens, HMB-PP, HMGB1 proteins, and melan-A, respectively), possibly turning weakly immunogenic tumors into strongly immunogenic tumors and promoting anti-tumor immunity. Thus, this approach may be the most effective way to achieve this objective. In general, however, there is a paucity of evidence in this regard; further work must be done to characterize the immune responses generated against neoantigens following intralesional therapy.

Another recurrent observation is that combined intralesional approaches (in the absence of systemic therapy) seem more effective than their individual treatments (Table 1). For example, combination of intralesional IL2 and ipilimumab [37], IL2 and imiquimod [56] (and with retinoid cream [33]), and BCG and imiquimod [29,30] all yielded higher response rates and overall outcomes than the monotherapies alone. The localized aspect of intralesional therapies is likely to benefit greatly from a multipronged approach, where additional drugs can be added intralesionally with an overall minor effect on adverse events, but a substantial effect on unbridling the host anti-tumor immune response. Indeed, when intralesional IL2 was given with systemic ipilimumab, severe irAEs were present and there were no objective responses to report [35], underpinning the importance of further investigation into intralesional delivery in advanced melanoma.

The cost of melanoma is projected to significantly increase in the near future [1,6]. A seldom-discussed point is the associated cost of treatment; both from a per-dose perspective and from associated costs of treatment (hospital fees, cost of managing toxicity, etc). Ipilimumab, for example, costs approximately $132,649 USD per regimen, with additional major associated costs for managing severe irAEs events and for hospital utilities [118]. Similarly, the cost of T-VEC therapy is approximately $362,033, putting the total cost of T-VEC and ipilimumab combination treatment at over $490,000 USD [119]. In stark contrast, a treatment regimen using IL2 would cost approximately $500 USD, which includes consult, biopsy, and follow-up costs [120]. Imiquimod, BCG, and retinoid creams all have costs that are miniscule in comparison to other regimens; ranging from a few dollars for BCG and retinoid cream to approximately $100 USD for imiquimod. Furthermore, many of these local and intralesional therapies, as discussed above, have mild adverse events, minimizing the cost of managing toxicities. Thus, from a cost–benefit perspective, it may be worthwhile to investigate using cheaper therapies, which have overall response rates that are equal to or greater than other, more expensive immunotherapies.

## Figures and Tables

**Table 1:** A summary of the clinical responses and adverse events observed for the different intralesional therapies in major studies published to date.

Intralesional Therapy	Citation	Total Enrollment	ORR (%) (*n*)	CR (%) (*n*)	PR (%) (*n*)	Adverse Events
**IL2**						
	Weide et al. [16]	48	90% (43)	69% (33)	21% (10)	Grade 1 and 2 only: injection site reaction, fever, nausea, fatigue, loss in appetite, dizziness
	Radny et al. [17]	24	83% (20)	62% (15)	21% (5)	Grade 1 and 2 only: local erythema, fever, flu-like symptoms, pain, fatigue, nausea, headache
	Hassan et al. [19]	31	87% (27)	32% (10)	55% (17)	Grade 1 and 2 only: fever, fatigue, chills, flu-like symptoms
	Boyd et al. [20]	39	82% (32)	51% (20)	31% (12)	Grade 1 and 2 only: injection site discomfort, fever, fatigue, chills
	Dehesa et al. * [21]	7	99.5%*	96%*	3.50%*	Grade 1 and 2 only: injection site discomfort, fever, fatigue, chills
**BCG**						
	Lieberman et al. [22]	6	67% (4)	50% (3)	17% (1)	Grade 1 and 2: fever, fatigue, child, malaise, nausea Grade 3 and 4: vomiting, local skin necrosis, hypotension, hepatic injury, granulomas, granulomatous replacement
	Yang et al. [25]	8	75% (6)	62.5% (5)	12.5% (1)	Not reported
	Cohen et al. [26]	4	0% (0)	0% (0)	0% (0)	Grade 3 and 4: severe hypotension, cardiovascular collapse, disseminated intravascular coagulation, acute kidney injury, hypokalemia, pulmonary edema
	Lardone et al. [27]	19	68% (13)	-	-	Not reported
**BCG and systemic ipilimumab**						
	Da Gama Guarte et al. [28]	5	0% (0)	0% (0)	0% (0)	Grade 1 and 2: injection site pain, rash, diarrhea Grade 3 and 4: small bowel obstruction, colitis, hepatitis
**BCG and imiquimod**						
	Kidner et al. [29]	9	67% (6)	56% (5)	11% (1)	Grade 1 and 2: injection site pain, injection site reaction, fever, chills
	Kibbi et al. [30]	3	100% (3)	100% (3)	0% (0)	Grade 1 and 2: fever, fatigue, injection site erythema, ulcer formation
**IL2 and imiquimod**						
	Green et al. * [31]	13	50.6%*	40.7%*	9.9%*	Grade 1 and 2: fever, flu-like symptoms, injection site reactions Grade 3 (1/13): rigors
**IL2, imiquimod, and retinoid cream**						
	Garcia et al. [32]	3	100% (3)	100% (3)	0% (0)	Grade 1 and 2: injection site reaction, chills, scarring, ulcer formation, erythema
	Shi et al. [33]	11	100% (11)	100% (11)	0% (0)	Grade 1 and 2: fever, fatigue, chills, nausea, injection site reaction, arthralgia, rigors, dermatitis
	Ogawa et al. [34]	4	100% (4)	100% (4)	0% (0)	Grade 1 and 2: injection site erythema
**IL2 and systemic ipilimumab**						
	Weide et al. [35]	15	0% (0)	0% (0)	0% (0)	Grade 1 and 2: flu-like symptoms, fatigue, injection site pain, rash Grade 3 and 4 (6/15): fatigue, pain, colitis
	Rafel-Shamsabadi et al. [36]	9	66% (6)	33% (3)	33% (3)	Grade 1 and 2: flu-like symptoms, fever, chills Grade 3 and 4 (1/9): peripheral polyneuropathy
**IL2 and intralesional ipilimumab**						
	Ray et al. [37]	12	66% (8)	58% (7)	8% (1)	Grade 1 and 2: flu-like symptoms, fatigue, chills, injection site pain Grade 3 and 4 (6/12): hyponatremia, ulceration
**L19-IL2**						
	Weide et al. [38]	24	50% (12)	25% (6)	25% (6)	Grade 1 and 2: injection site pain, fatigue, erythema, local edema
**T-VEC**						
	Senzer et al. [39]	50	26% (13)	16% (8)	10% (5)	Grade 1 and 2: flu-like symptoms, fever, fatigue, chills
	Andtbacka et al. (2015) [40]	295	26% (78)	11% (32)	15% (46)	Grade 1 and 2: flu-like symptoms, fever, fatigue, chills, nausea Grade 3 and 4 (39/292): severe fatigue, vomiting, pain, cellulitis, peripheral edema
	Perez et al. (2018) [41]	23	56% (13)	43% (10)	13% (3)	Grade 1 and 2: flu-like symptoms, fever, chills Grade 3 and 4 (3/23): cellulitis, bleeding ulceration
	Louie et al. [42]	80	56% (45)	39% (31)	17% (14)	Grade 1 and 2: flu-like symptoms, fever, chills
	Zhou et al. [43]	40	48% (19)	43% (17)	5% (2)	Grade 1 and 2: fever, fatigue, injection site pain Grade 3 and 4 (3/40): cellulitis, neurological changes, periorbital edema
	Perez et al. (2019) [44]	76	-	20% (15)	-	Grade 1 and 2: flu-like symptoms, fever, fatigue, chills Grade 3 and 4 (5/76): ulceration, cellulitis, injection site pain
	Chesney et al. [45]	41	-	12% (5)	-	Grade 1 and 2: flu-like symptoms, fever, fatigue, chills, nausea Grade 3 and 4 (10/41): severe rigors, severe fever, injection site infection, vomiting, hyperhidrosis
	Andtbacka et al. (2016) [46]	61	48% (29)	30% (18)	18% (11)	Grade 1 and 2: flu-like symptoms, fever, fatigue, chills, nausea
**T-VEC and systemic ipilimumab**						
	Puzanov et al. [47]	18	50% (9)	22% (4)	28% (5)	Grade 1 and 2: fever, fatigue, chills, diarrhea Grade 3 and 4 (5/18): severe fever, severe nausea, dehydration, vomiting, increased lipase and amylase
**T-VEC and systemic pembrolizumab**						
	Long et al. [48]	21	47% (10)	14% (3)	33% (7)	Grade 1 and 2: fever, fatigue, chills Grade 3 and 4 (7/21): details not reported
**Rose Bengal (PV-10)**						
	Thompson et al. (2008) [49]	11	54% (6)	27% (3)	27% (3)	Grade 1 and 2: injection site pain, pruritis, local erythema
	Read et al. [50]	45	86% (39)	42% (19)	44% (20)	Grade 1 and 2: injection site pain, local edema, local erythema Grade 3 and 4 (3/45): ulceration, cellulitis, photosensitivity reaction
	Lippey et al. [51]	19	52% (10)	26% (5)	26% (5)	Grade 1 and 2: injection site pain, local edema, local erythema Grade 3 and 4 (1/19): cellulitis
	Thompson et al. (2015) [52]	80	51% (41)	26% (21)	25% (20)	Grade 1 and 2: injection site pain, local edema, vesicles, local edema, pruritis Grade 3 and 4 (12/80): severe pain, vesicles, local edema, peripheral edema, dysphagia, photosensitivity reaction

* Only reported on lesion response rates and not individual response rates. ORR = overall response rate; CR = complete response; PR = partial response; IL2 = interleukin-2; BCG = Bacillus Calmette-Guerin; T-VEC = Talimogene Laherparepvec.

**Table 2:** A summary of the proposed immunomodulatory mechanism of action of the different intralesional therapies discussed.

Intralesional Therapy	Proposed Mechanism of Action
IL2	Infiltration of CD3^+^ cell infiltrate rich with monocytes, macrophages, and melanophages [17]. Possibly immune-induced cell death of tumor cells via caspase 3-mediated apoptosis [17]. Specific anti-tumor immune response was identified, with anti-MAGEA3 T cells present in the infiltrate [16].
BCG	Both injected and uninjected tumors rich with a CD3^+^ cell infiltrate, with γδT cells recruited through CXCL9, CXCL10, and CXCL11 [25]. Effector γδT cells, primarily of the Vδ2 subtype, which are activated by mycobacterial molecules (HMG-PP) produce IFNγ, TNFα, TNFβ, and IL15, promoting a potent antitumor innate-like immune response. There is a noted absence of α/β T cells [25]. BCG injection induces immune plasticity in M2 macrophages, which decrease pro-tumorigenic cytokine production (such as IL10) and increase anti-tumorogenic cytokine production (such as IL12), bolstering a Th1 response. Remodeled M2 macrophages stimulate CD4^+^ T cells to produce IFNγ, and CD8^+^ T cells to produce granzyme B [27].
BCG and imiquimod	TLR7 agonism via imiquimod potentiates recruitment of CD4^+^ and CD8^+^ T cells [53]. Combination treatment promotes recruitment of B cells and plasmacytoid dendritic cells [54], and IFNγ [54], IFNα/β [54], IL6 [55], and IL12 [55] are present in the tumor microenvironment.
IL2 and imiquimod	TLR7 agonism sensitizes the immune response to IL2 injection, leading to a robust Th1 anti-tumor response with an increase in CD4^+^ and CD8^+^ T cells. A downregulation of pro-tumor immune cytokines (such as IL5) and upregulation of anti-tumor cytokines (such as IFNγ) is observed [56].
IL2, imiquimod, and retinoid cream	Addition of retinoid signaling further increases anti-tumor Th1 cytokines IL6, TNFα, and IL2Rα. Th1 transcription factors T-bet, STAT1, and STAT4 are increased, while Th2 and Th17 transcription factors GATA3 and RORC are decreased [57]. Anti-melanocyte immune response (vitiligo) is noted, with presence of immune effector T cells and memory T cells in the infiltrate [57].
IL2 and intralesional ipilimumab	Despite intralesional treatment, systemic increases in IFNγ, granzyme B, T-bet, and perforin were observed in those with objective responses [37]. Possible depletion of local T regulatory cells [58].
T-VEC	Increases infiltrating CD8^+^ T cells expressing perforin and granzyme B, as well as memory effector T cell phenotypes [59]. Inhibits intratumoral immunosuppressive cells (T regs and MDSCs) [59]. Promotes systemic immunity via upregulation of IFNγ-producing anti-Melan-A CD8^+^ T cells [59].
T-VEC and systemic pembrolizumab	Addition of systemic checkpoint inhibitor further potentiated anti-tumor immune responses, by increasing IFNγ-producing CD8^+^ T cells, as well as B cells and memory effector T cells. This effect was observed in both injected and uninjected lesions [60].
Rose Bengal (PV-10)	Promotes proliferation of activated CD8^+^ T cells and chemotaxis to draining lymph nodes, with increases in memory effector T cell subsets systemically [61]. Tumor antigen uptake into DCs is increased, as is DC migration to draining lymph nodes [61]. Induces necrosis in melanoma cells; HMGB1 is released and taken up by DCs, further contributing to a specific anti-tumor immune response [61].
